# Organ-on-a-Chip Platforms for Drug Screening and Delivery in Tumor Cells: A Systematic Review

**DOI:** 10.3390/cancers14040935

**Published:** 2022-02-13

**Authors:** Inês M. Gonçalves, Violeta Carvalho, Raquel O. Rodrigues, Diana Pinho, Senhorinha F. C. F. Teixeira, Ana Moita, Takeshi Hori, Hirokazu Kaji, Rui Lima, Graça Minas

**Affiliations:** 1 METRICS, University of Minho, Alameda da Universidade, 4800-058 Guimarães, Portugal; id9385@alunos.uminho.pt (I.M.G.); violeta.carvalho@dem.uminho.pt (V.C.); rl@dem.uminho.pt (R.L.); 2 IN+—Center for Innovation, Technology and Policy Research, Instituto Superior Técnico, Universidade de Lisboa, Avenida Rovisco Pais, 1049-001 Lisboa, Portugal; anamoita@tecnico.ulisboa.pt; 3 Center for MicroElectromechanical Systems (CMEMS-UMinho), Campus de Azurém, University of Minho, 4800-058 Guimarães, Portugal; diana.pinho@inl.int; 4 ALGORITMI Center, Campus de Azurém, University of Minho, 4800-058 Guimarães, Portugal; st@dps.uminho.pt; 5 LABBELS-Associate Laboratory, Braga/Guimarães, 4806-909 Guimarães, Portugal; 6 CINAMIL—Centro de Investigação Desenvolvimento e Inovação da Academia Militar, Academia Militar, Instituto Universitário Militar, Rua Gomes Freire, 1169-203 Lisboa, Portugal; 7 Department of Biomechanics, Institute of Biomaterials and Bioengineering (IBB), Tokyo Medical and Dental University (TMDU), Chiyoda, Tokyo 101-0062, Japan; hori.bmc@tmd.ac.jp (T.H.); kaji.bmc@tmd.ac.jp (H.K.); 8 CEFT, Faculty of Engineering, University of Porto, Rua Dr. Roberto Frias, 4200-465 Porto, Portugal

**Keywords:** cancer cells, hard tissues and organs, organoids, microfluidics, organ-on-a-chip, microbioreactor, drug screening, cell culture, drug delivery, nanoparticles

## Abstract

**Simple Summary:**

Cancer is one of the diseases with a high mortality rate worldwide. Of the current strategies to study new diagnostic and treating tools, organs-on-chip are quite promising regarding the achievement of more personalized medicine. In this work, 75 out of 820 of the most recent published scientific articles were selected and analyzed through a systematic process. The selected articles present the different microfluidic platforms where cell culture was introduced and was used for the evaluation of cancer treatments efficacy and/or toxicity.

**Abstract:**

The development of cancer models that rectify the simplicity of monolayer or static cell cultures physiologic microenvironment and, at the same time, replicate the human system more accurately than animal models has been a challenge in biomedical research. Organ-on-a-chip (OoC) devices are a solution that has been explored over the last decade. The combination of microfluidics and cell culture allows the design of a dynamic microenvironment suitable for the evaluation of treatments’ efficacy and effects, closer to the response observed in patients. This systematic review sums the studies from the last decade, where OoC with cancer cell cultures were used for drug screening assays. The studies were selected from three databases and analyzed following the research guidelines for systematic reviews proposed by PRISMA. In the selected studies, several types of cancer cells were evaluated, and the majority of treatments tested were standard chemotherapeutic drugs. Some studies reported higher drug resistance of the cultures on the OoC devices than on 2D cultures, which indicates the better resemblance to in vivo conditions of the former. Several studies also included the replication of the microvasculature or the combination of different cell cultures. The presence of vasculature can influence positively or negatively the drug efficacy since it contributes to a greater diffusion of the drug and also oxygen and nutrients. Co-cultures with liver cells contributed to the evaluation of the systemic toxicity of some drugs metabolites. Nevertheless, few studies used patient cells for the drug screening assays.

## 1. Introduction

The development of new tools for diagnostic and treatment is a process of great importance due to the complexity and variety of diseases that affect the population. Nevertheless, the process is time-consuming, owing to the many steps it takes to reach the final product, apart from being quite expensive [1]. Furthermore, the development of new tools with compounds that present great potential can be hindered by the difficulty in performing some of the required steps. For instance, nanoparticles have shown potential as drug carriers or antibacterial agents, but they have difficulty reaching clinical tests due to the complexity in ascertaining their interaction with specific cellular pathways in specific environments [2]. It is of general agreement in the scientific community that the current models used in the preliminary tests of new diagnostic or treatment techniques do not properly represent the tissue microenvironment found in humans. The most common in vivo models are transgenic mice that are immunocompromised and have specific gene mutations, depending on the assay, which can create a biased response in comparison with humans. Additionally, and despite similarities in the manifestation of the disease, those models are unable to report some of the molecular mechanisms, for example, how individual cells (i.e., pathogenic cells, organ cells, immune cells) interact in the physiologic environment [3,4]. Animal models also evoke ethical problems, which can be surpassed by in vitro bioplatforms. So, the research of new in vitro preclinical platforms is being focused on the replication of the in vivo tissue- and organ-level physiological interactions and manifestations of the disease [3,5].

During the last 20 years, the progress of microfluidic systems has attracted several researchers and has been applied in several fields ranging from renewable energy to nanomedicine [6,7,8,9]. In the field of biomedical applications, novel advanced microfluidic devices comprising biomodels have been developed to recreate the microenvironments and cellular functions and interactions present in certain organs. Those systems were designated organ-on-a-chip (OoC) [6,10,11]. OoC is a device that combines microfluidics and cell culture, made of glass, polymers, e/or plastic to be optically clear and, as mentioned before, is able to recapitulate the tissue- and organ-level pathophysiology present in vivo. Since they were made using the same micromanufacturing methods used for computer microchip fabrication, they attained the designation of ‘chip’ and were defined as a specific field after the publication of the Ingber team [12], on the lung-on-a-chip, in 2010. Among the advantages of these new bioplatforms, the cell culture can be maintained for long periods of time due to the flow of culture medium throughout the microchannels, and it can be monitored by connecting different types of equipment, such as microscopes, incubators, pumps, or sensors (temperature, pH) to the chip [4,13,14]. Such devices are of great interest for the evaluation of the effect and toxicity of new drugs since they can better recapitulate the human biochemical environment than some of the animal models that are used [15,16,17].

One of the top leading causes of death worldwide, according to the World Health Organization, is cancer [18]. The development of new and personalized treatments for the patients is hindered by the high cost and the tardiness to transition to clinical trials [19]. The development of new anticancer drugs requires the assessment of their effects on cells in a high throughput and cost/time effective fashion. Monolayer cell cultures in 2D are a standard tool for drug screening since they are easy to establish and cost-effective systems [20]. However, cells cultured in 2D systems do not present the same interactions that are seen in 3D configurations, which leads to a non-accurate representation of the human in vivo response. Thus, the prediction of drug effects on those systems can be affected by such inaccuracy, leading to ineffective drugs being used in further studies [21]. Three-dimensional (3D) models for cell culture, such as organoids and spheroids, contributed to better mimicking the in vitro cell–cell interactions that occur among cancer cells and between cancer and healthy cells. In the case of cancer cells, it is possible to reproduce the formation of a necrotic core when the spheroid is big enough to create a gradient of nutrients and oxygen. In recent studies, 3D cultures technology has been used to grow healthy and cancer tissue models for the evaluation and development of anticancer drugs. Cells in 2D cultures do not present the same cell–cell or cell–environment interactions as cells in 3D cultures. Therefore, the differentiation, proliferation, and organization of the cells on the later cultures is a better replication of the physiological conditions and a better recapitulation of the tumor microenvironment features [20,21,22]. Those are important features needed for dynamic drug testing and for the understanding of the molecular pathways that drive the tumor response to a drug. The developed biomimetic models such as spheroids and organoids have already demonstrated in static or dynamic conditions that they allow direct control of biomechanical, biochemical, and biophysical microenvironments and the use of novel biomaterials that present extracellular matrix (ECM) elements can contribute to the enhancement of the cellular activity [21]. The use of this type of cell culture contributes to the reduction of ineffective anticancer drugs to be tested for in vivo assays [22]. Furthermore, the use of patient-derived cells also contributes to the evaluation of disease progression and the study of specific biomarkers to develop personalized treatments and the understanding of drug resistance [20]. Nevertheless, by themselves, those 3D cellular structures, without the dynamic flow, do not recreate the influence of pressure and stress caused by the fluids and tissues in the environment, nor the supply of nutrients, cells, and other compounds by the blood [4]. This challenge is overpassed by the new technology ‘tumor-on-a-chip’ that merges 3D cancer models and microfluidics. Therefore, it is a promising tool to better simulate the cancer environment found in vivo and a promising tool that can be used for drug screening and also to study events such as the formation and spreading of metastasis [19].

In this review, a systematic search based on the Preferred Reporting Items for Systematic Reviews and Meta-Analyses (PRISMA) guidelines was performed in three databases using a selected set of keywords. The results of the search were revised and selected by two of the authors in accordance with the criteria that were defined for this review. The selected studies were then divided following the type of tumor cell line used and their main conclusions reported herein. Overall, the use of OoC devices presents several advantages when compared to 2D cultures. The combination of different organ modules on the chip also contributes to a more accurate evaluation of the drug effects.

## 2. Methods

This work was based on the research guidelines for systematic reviews proposed by PRISMA [23,24], with PROSPERO registration number 303752.

### 2.1. Data Sources and Search Strategy

The search was performed in three different databases: ScienceDirect, PubMed, and Scopus. The search on all bases was performed until the 1st of March of 2021. The search string used was (“organoids” OR “organ-on-a-chip” OR “organ on chip”) AND (“cancer cell” OR “tumor cell”) AND (”drug screening” OR “drug delivery” OR “cancer drug” OR “cancer treatment”).

### 2.2. Validity Assessment

Review articles, conference papers, short-communications, and non-English written articles were removed from the search results, either manually or using the filters from the database. After the elimination of the duplicates, the articles were selected based on the relevance of their title in the context of this review. Further screening was performed to evaluate which paper presented the defined inclusion or exclusion criteria presented below. To avoid biases, two authors performed the screening and selection of the research papers separately and then compared the classifications. Disagreements or doubts regarding the classification were solved by a third author.

### 2.3. Inclusion and Exclusion Criteria

The studies included in this review followed the criteria:

- Published in the last 10 years;
- The cell lines used corresponded to cancer cells;
- Use of microfluidic platforms or organs-on-chip in cancer studies;
- The 3D models allowed the performance of drug screening;
- Use of 3D cell culture models to test a new or standard cancer treatment.

The studies that did not present the mentioned criteria were excluded from this study.

## 3. Results and Discussion

### 3.1. Study Selection

**Figure 1:**
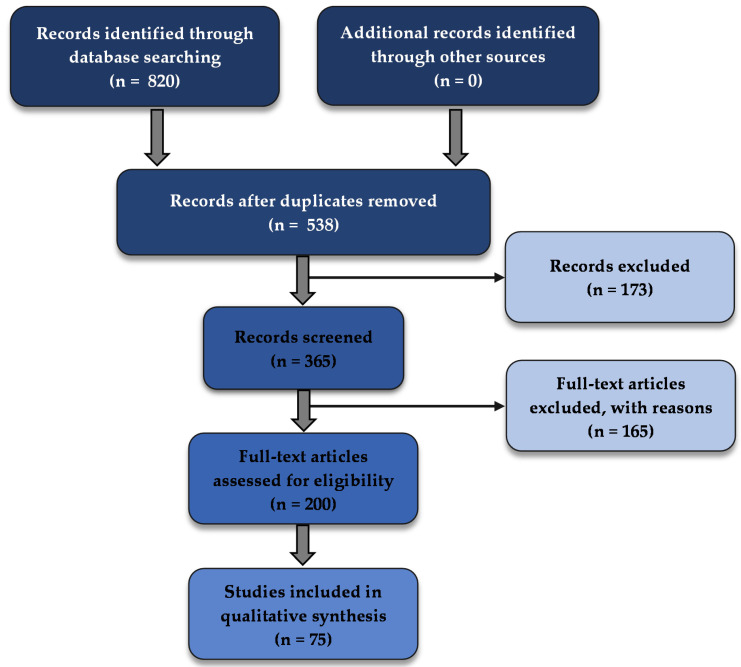
PRISMA flow diagram of the systematic selection of articles.

In the Science Direct database, a total of 520 results were obtained, of which 69 were research articles; from the PubMed database, six results were obtained, of which six were research articles; and from the Scopus database, 1779 results were obtained, of which 745 where research articles. No other articles from other sources were added. After the duplicates were removed, a total of 365 articles were selected from the obtained 820. Of those records, 200 were eligible for this study, and after the final screening by evaluating the relevant sections of the selected records was performed. The final number of the selected articles was 75. A schematic representation of the selection is presented in Figure 1.

**Figure 2:**
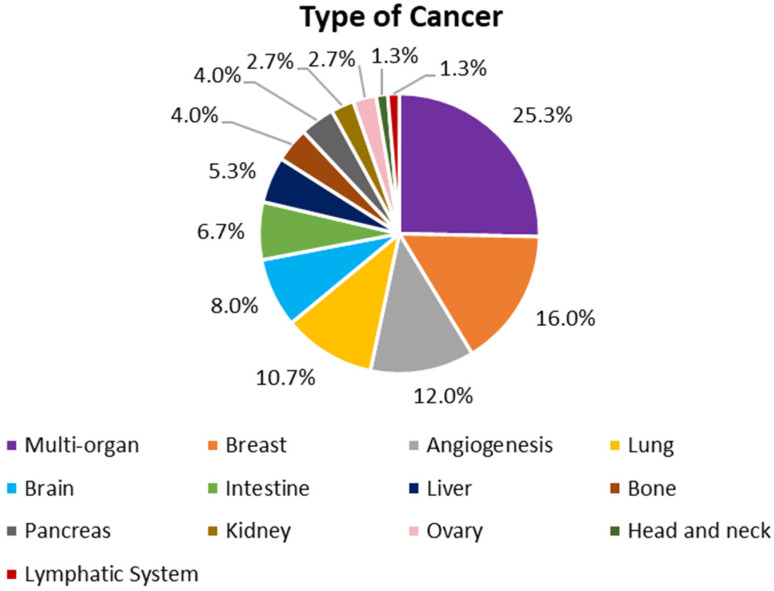
Types of cancer replicated in the OoC of the selected articles of this review.

The selected articles were divided in accordance with the type of cancer model the research group replicated in the OoC. Some groups focused on the study of blood vessel formation angiogenesis on the area surrounding a tumor. A great number of studies combined more than one organ to simultaneously evaluate the effect of anticancer drugs on different cancer types or to evaluate the toxic effects of those drugs on healthy organs. The percentage of studies for each group is presented in Figure 2.

### 3.2. Organ Models

#### 3.2.1. Brain and Nervous System

**Figure 3:**
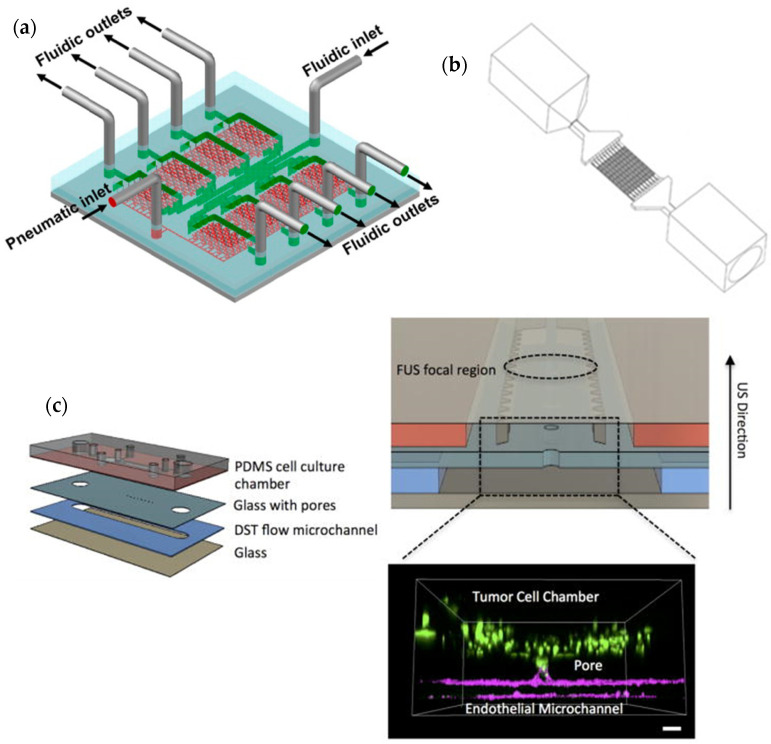
(**a**) Schematic representation of a microfluidic device for repeatable 3D culture of glioblastoma cells used by Liu et al. [26]. Reprinted with permission from [26]; (**b**) Perspective view of the entire microfluidic device developed by Tricinci et al. [28] for BBB mimicking and glioblastoma spheroids culture. Reprinted with permission from [28]; (**c**) Different layers of the microfluidic device for glioblastoma 3D cell culture proposed by Zervantonakis and Arvanitis [29]. Reprinted with permission from [29].

Ma et al. [25] replicated the 3D in vivo microenvironment of glioblastoma using a collagen matrix embedded with self-assembled glioblastoma spheroids integrated on a microfluidic device. The efficacy of resveratrol (Res) as an anti-tumoral agent was tested on the device and compared to the effect of temozolomide (TMZ), as well as the combination of both drugs. The results indicated that Res alone induced more cell death than TMZ or the combination of TMZ and Res. Liu et al. [26] manufactured a microfluidic device to culture glioblastoma cells under different culture conditions, as presented in Figure 3a. The authors verified that the stability of the culture was maintained for over a month period. The device was also used for the evaluation of the anticancer effects of the chemotherapeutic drugs vincristine (VCR) and bleomycin (BLM) in six different concentrations. The higher concentration (100 μg/mL) of both drugs led to the death of tumor cells and a reduction of the tumor size after four days of treatment. VCR was more effective than BLM, killing about 80% of the cells and reducing the tumor size by 49%. The authors also performed similar assays using 2D cell culture and noted a greater drug resistance in the 3D culture. Ayuso et al. [27] recreated the microenvironment of a tumor for real-time monitoring of glucose and oxygen concentration, as well as reactive oxygen species (ROS) generation, cell proliferation, and apoptosis. Glioblastoma cancer cells were cultured in a hydrogel on the chip, and the response to TMZ treatment was evaluated and also compared to a 2D culture. The viability reduction effect of the drug on the cancer cells was less pronounced on the 3D culture, being the levels of cell death only higher in the most proliferative and oxygenated areas of the chip. The effect of the drug tirapazamine (TPZ) was also tested. The greater cell death rates were registered in the innermost hypoxic areas of the culture.

Yi et al. [30] developed a glioblastoma-on-a-chip using patient-derived tumor cells, decellularized extracellular matrix from brain tissue, and vascular endothelial cells. The authors reported that clinically observed features of the patients were replicated on the chip, such as the resistance to the treatment with chemoradiation and TMZ. Other treatments, namely cisplatin (CIS), KU60019 (KU)39, and two types of inhibitors of the DNA repair mechanism, and their combinations were also tested on two types of tumorous cells from the same patient. For all the treatments tested, a reduction of cancer cells was reported, although one type was more susceptible than the other.

Tricinci et al. [28] mimicked the microenvironment of a brain tumor using a 3D-printed model, presented in Figure 3b, that included a co-culture of the two cell types that form the blood–brain barrier (BBB) and glioblastoma spheroids. The permeability of the BBB was accessed using fluorescent dextran. Antibody-functionalized nutlin-loaded nanostructured lipid carriers were tested for cancer treatment. The authors reported an intake of the particles by about 70% of the glioblastoma cells.

Zervantonakis and Arvanitis [29] investigated the potential of the combination of focused ultrasound (FUS) and nanomedicine drugs to improve the treatment of glioblastoma. For that purpose, an acoustofluidic platform (Figure 3c) was developed to culture glioblastoma cells in a 3D environment and to apply the FUS treatment. Nanoparticles of doxorubicin (DOX) encapsulated in temperature-sensitive liposomes were used, and the heat generated by FUS contributed to a higher uptake of the drug by the cells resulting in an increase in cell death and a reduction of cell proliferation when compared to the isolated treatments.

#### 3.2.2. Head and Neck

Kennedy et al. [31] developed a tumor-on-a-chip to recapitulate the microvascular flow and diffusion of a head and neck squamous cell carcinoma that occurs in vivo. Slices of tumorous tissue derived from the patient’s biopsies were cultured on the chip and subjected to treatment by irradiation and drug administration (CIS). The irradiation was performed in relevant doses (5 × 2 Gy) and led to an increase in DNA damage apoptosis and reduced the proliferation of the cancer cells. The addition of CIS further increased the apoptosis rate.

#### 3.2.3. Bone

**Figure 4:**
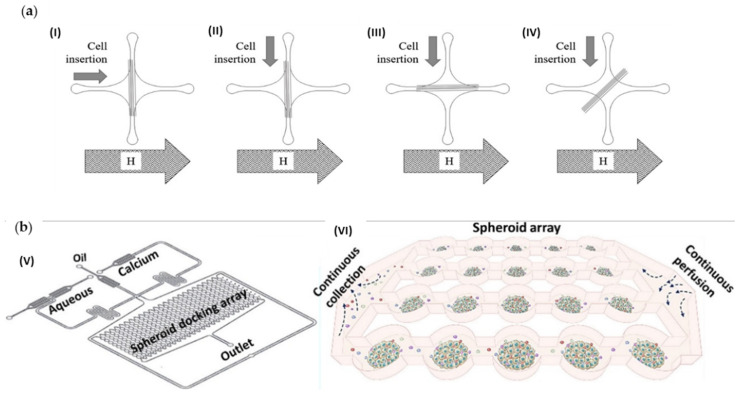
(**a**) Schematic representation of the microfluidic device developed by Mitxelena-Iribarren et al. [34] with microwires position perpendicular (I), (II), parallel (III), or diagonal (IV) regarding the magnetic field and orthogonal (I), (II), longitudinal (II) and diagonal (IV)) cell insertion regarding the microwires. Reprinted with permission from [34]. (**b**) Sabhachandani et al. [35] proposed the microfluidic device represented in (V) and (VI) representation of the microarray for spheroid cell culture. Reprinted with permission from [35].

Mitxelena-Iribarren et al. [32] optimized a microfluidic device for the culture and proliferation of osteosarcoma cell lines to evaluate the effects of different treatments on the cells. The authors compared the use of the chemotherapy agent methotrexate (MTX) by itself with the same agent encapsulated with lipid nanoparticles. Seventy-two hours after treatment with MTX-lipid nanoparticles, a reduction of 40% of the cell population was reported by the researchers. On the other hand, after the treatment with free MTX, the cell population increased. The authors also noted that no sedimentation of the particles was registered. In another study [33], the same group developed another microfluidic platform with microstructures to simultaneously compare the effect of free MTX, MTX-loaded Lecithin (LEC)-Tween 80 nanoparticles and MTX-loaded LEC-PVA nanoparticles on osteosarcoma cells. The MTX-loaded LEC-Tween nanoparticles reduced the cell population to 2.3%, while the LEC-PVA nanoparticles reduced to less than 20%, 72 h after treatment. The authors also noted that the microfluidic platform contributed to a faster internalization of the particles by the cells when compared to plain microfluidic platforms, and, consequently, the effectiveness of the tested drugs was faster than conventional methods. The same group also assessed the efficacy of Fe_77_B_10_Si_10_C_3_ glass-coated amorphous magnetic microwires for hyperthermia treatment of osteosarcoma (Figure 4a) [34]. A reduction of up to 89% of the osteosarcoma cell population was registered after about 1 h of subjecting the microwires in the cell culture to different magnetic fields. Conversely, the hyperthermia treatment had little effect on healthy cells.

#### 3.2.4. Lymphatic System

Sabhachandani et al. [35] developed a microfluidic platform, presented in Figure 4b, for the study of a subtype of Non-Hodgkin lymphoma named diffuse large B cell lymphoma (DLBCL). Spheroids of fibroblasts, lymphocytes, and DLBCL cells were cultured on a hydrogel inside the device, and the cellular dynamic activity was analyzed. The response to the drug lenalidomide was also evaluated by the authors using the 3D culture on the microfluidic platform, as well as on a 2D culture. Cell death and reduction of the proliferation of DLBCL spheroids and cells in 2D culture after treatment was higher than in the 3D culture. The other cell types were also affected by the treatment on the 2D culture. This suggests that cells have different mechanisms for drug transport and diffusion when in 2D or in 3D cultures. Furthermore, the presence of an ECM might contribute to the increased resistance of the cells to the drug.

#### 3.2.5. Angiogenesis

Liu et al. [36] simulated the 3D tumor-microvascular structure on a microfluidic device to evaluate the therapeutic effect of the antioxidants α-lipoic acid, catechins and ascorbic acid on glioma cells. The evaluation was performed based on the levels of ROS and glutathione (GSH). The clearance of ROS was more prominent on the cancer cells than on the endothelial cells forming the vessels, being the α-lipoic acid the most effective agent. The levels of ROS were reduced, while the levels of GSH increased with the increasing concentration of the antioxidant agent. Ko et al. [37] presented a platform for spheroid culture made by injection molding of polystyrene. A 3D perfusable blood vessel network was also incorporated to better recreate the tumor microenvironment. The anti-angiogenic effect of the cancer drugs sunitinib and bevacizumab was evaluated on the device with a co-culture of glioma cells and endothelial cells. The sprout’s number and length were reduced, as well as the vascular network area, after the treatment with each of the drugs. The effect of cetuximab was also evaluated, but no significant differences between the treatment with the drug and the control were reported.

**Figure 5:**
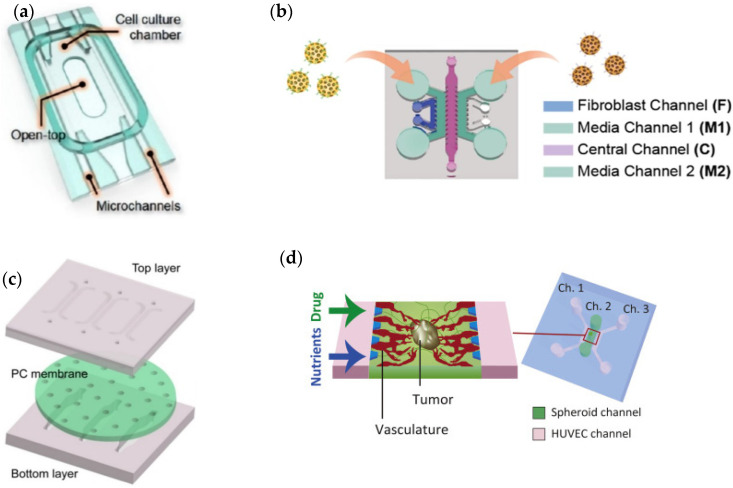
(**a**) Paek et al. [38] proposed the a device comprising a cell culture chamber with a top opening and two parallel microchannels used for controlled vascular perfusion. Adapted with permission from [38]; (**b**) Lee et al. [39] design a chip for angiogenesis assay. Adapted with permission from [39]; (**c**) a microfluidic chip design by Wang et al. [40] constructed with top and bottom layers separated by PC membrane. Adapted with permission from [40]; (**d**) Microfluidic platform to recapitulate in vivo tumor microenvironments, proposed by Nashimoto et al. [41]. Adapted with permission from [41].

Paek et al. [38] recreated on a microfluidic device the vascular networks that can be observed in a human lung adenocarcinoma microenvironment (Figure 5a). The authors used the device for the study of the effect of the chemotherapeutic drug paclitaxel (PTX) on the cancer spheroids and vasculature. After two days of perfusion, a 20% decrease in cell viability in the spheroids was registered. The dead cells were found in a spatially heterogeneous distribution and near the center of the spheroids due to the vasculature system contribution to spreading the drug to the inner parts of the spheroids. On the other hand, the authors also reported changes in the vasculature system, such as reduction of average vessel diameter, length, and density, which might be correlated with the negative cardiac side-effects of the drug.

Lee et al. [39] designed a microfluidic platform to recapitulate the angiogenic formation that occurs in tumors in vivo and to evaluate the efficacy of an RNAi-based anti-angiogenic nanomedicine, as shown in Figure 5b. The selected target for the siRNA was siVEGF (vascular endothelial growth factor) or siVEGFR (vascular endothelial growth factor), and the carrier was a mesoporous silica nanoparticle. Firstly, the authors evaluated the toxicity of the nanoparticles on healthy cells in 2D culture. Then, three different cell types, HepG2, SW620, and A549, were selected to evaluate the drug efficacy using the advanced microfluidic device. The treatment had little to no effect on the SW620 and A549 cell lines since they naturally present a low degree of angiogenesis. On the other hand, the anti-angiogenic effect was prominent on the HepG2 cell line since it is a hyper-angiogenic cancer type.

Wang et al. [40] constructed a microfluidic chip that simulates the tumor-vascular microenvironment (Figure 5c). Hepatic and breast tumor cell lines were cultured on the device along with endothelial cells and fibroblasts. The effect of DOX was evaluated. The authors reported an increase in the cancer cells viability when cultured with endothelial cells and fibroblasts than without those cells. Consequently, the authors reported that the dynamics of the physiological environment affect the drug efficacy. Nashimoto et al. [41] presented a tumor-on-a-chip platform with a perfusable vascular network for the study of tumor progression as schematized in Figure 5d. The authors evaluated the effects of the drug PTX on single, co- and tri-cultures of breast cancer, lung cancer, and colon cancer cell lines. Under static conditions, the drug led to the reduction of the size of the tumor spheroids with increasing concentration. On the other hand, the effect of the drug on the culture on the chip was not dose-dependent and was less pronounced than the previous condition. The difference was possibly due to the extra supply of oxygen and nutrients by the vascular network.

Liu et al. [42] developed a microfluidic model for the seeding of cancer cells and the replication of the perfusable vascular networks surrounding the tumor. The anticancer drugs sorafenib, fluorouracil, and VCR were tested on the device on perfusable and non-perfusable colon cancer cultures. The authors reported that for low concentrations of the drugs, the cancer spheroids would grow again after the treatment, but the reduction of the vascular network would remain. The cytotoxic effect of the drugs was greater on the perfusable culture, possibly due to the drug reaching more regions of the tumor.

Sobrino et al. [43] described a tumor-on-a-chip device that recreates the microenvironment of a tumor, including the vascular system surrounding the cells. Different cancer cells were cultured on the platform, and their response to standard drug treatment was evaluated. In breast and colorectal cancer cells, a regression of tumor growth after treatment was registered. Treatments with the drugs apatinib and vandetanib did not reduce the vasculature, while linifanib and cabozantinib did, likely due to the latter targeting effect being not only VEGFRs but also platelet-derived growth factor receptors and the angiopoietin receptor Tie2. Phan et al. [44] described a microfluidic platform for the establishment of vascularized micro-organs to evaluate the anticancer and anti-angiogenic effects of cancer drugs. The authors used FDA-approved anticancer drugs on endothelial, lung fibroblasts, and colorectal cancer cells cultured on the chip. Some of the drugs reduced vascular formation, others reduced tumor growth, and others had both effects. Interestingly, the effectiveness of some of the tested drugs was superior to the registered in 2D monolayer cultures, while the opposite was noticed on others.

#### 3.2.6. Colorectal

**Figure 6:**
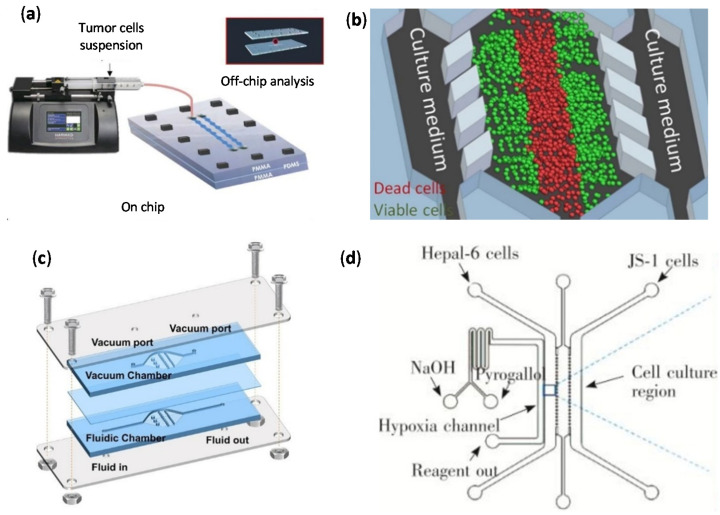
(**a**) Pitingolo et al. [45] developed a cell culture on chip by using a syringe pump to control the flow injection. Adapted with permission from [45]; (**b**) Schematic representation of the culture medium perfusion through the device used by Ayuso et al. [27]. Adapted with permission from [27]; (**c**) Liang et al. [48] developed the bubble trap device schematized. Adapted from [48]; (**d**) Representative diagram of the co-culture chip used by Sun et al. [49] to evaluate the anticancer effects on hepatic healthy and cancer cells. Adapted with permission from [49].

Pitingolo et al. [45] designed a tumor-on-chip for colorectal carcinoma cell growth and drug response evaluation. The device illustrated in Figure 6a comprises a set of microwells connected through microchannels and bonded by a magnetic system, making it possible to re-open. On the wells, the researchers cultured colorectal cancer cells and evaluated the cytotoxic activity of camptothecin (CPT) in different concentrations (100 μM and 400 μM). An increase in cell death and a decrease in the spheroids’ size was registered after the treatment with the drug, being the effects more evident with the higher dosage used. Komen et al. [46] presented a microfluidic device with a culture chamber for the seeding of colorectal cancer cells and a drug-dosing channel for the controlled administration of anticancer drugs. The chamber and the channel are separated by a transparent membrane. The authors tested the static and dynamic administration of oxaliplatin to the cancer cells. The cell growth was reduced in a dose-sensitive manner compared to the untreated cultures. Greater efficacy was reported when continuously administrating the drug than when exposing the cells to the maximum concentration. Ayuso et al. [27] recreated the microenvironment of a tumor for real-time monitoring of glucose and oxygen concentration, as well as ROS generation, cell proliferation, and apoptosis (Figure 6b). Colorectal cancer cell spheroids were cultured on the chip, and the response to DOX treatment was evaluated. The drug caused the death of the cancer cells, with the effect more prominent near the lateral microchannels and less far from the microchannel in the hypoxic region. Carvalho et al. [47] described a tumor-on-a-chip model to evaluate the efficacy of drug-loaded nanoparticles in the treatment of colorectal carcinoma. The cell culture was exposed to gemcitabine (GEM)-loaded CMCht/PAMAM nanoparticles over a period of five days. The authors reported an increase in cell death throughout the days, indicating the progressive release of the drug from the particles.

Rajan et al. [50] constructed a photopatterned microfluidic device for the study of a 3D culture of colon carcinoma cells. The viability and invasion dynamics of the cells were evaluated after the exposure to normal media and two anticancer drugs, anti-migratory Marimastat and anti-proliferative 5-fluorouracil (5-FU), separately. After the ten days treatment with 5-FU, a strong reduction of the cell viability was registered, as well as a reduction of the number of migrating cells, independent of the dose. However, the distance traveled by the migrating cells was not affected by the drug exposure. On the other hand, Marimastat reduced the number of migrating cells and the migrating distance in a dose-dependent manner.

#### 3.2.7. Pancreas

Beer et al. [51] used a commercial cyclic olefin polymer chamber (HepaChip^®^) to test the potential to study the growth and drug resistance of different pancreatic ductal adenocarcinoma (PDAC) human cell lines. The authors analyzed the anticancer effects of CIS in the 3D culture on the chip and compared it to a 2D culture and an in vivo test in immunocompromised mice. After 72 h of treatment, the cell viability was greatly reduced (about 80%) on both 2D and 3D cultures. As expected, the half-maximal inhibitory concentration (IC_50_) value for the 3D culture (14.6 ± 1.6 μM) was higher than the value for the 2D culture (3.25 ± 0.2 μM). In the mice, the tumor growth was completely inhibited after ten days. The authors also verified if the adsorption of the drug by the walls of the microchannels could be the cause for the need for higher doses in the 3D culture. However, no significant adsorption was noted, being the drug fully recovered after 1 h of perfusion.

Moon et al. [52] developed a microfluidic device to recreate the heterogeneity present in the intra-tumoral environment in vivo. The authors used PDAC cell lines with different genotypes and phenotypes and tested the resistance to the drug GEM. Though the drug affected the cell viability, the dose with more prominent effects was higher when compared to 2D cultures of the same cell lines. The increased resistance to the drug could be correlated with the cell–cell interactions since a greater increase in resistance was registered in co-cultures than in single cultures. Kramer et al. [53] also presented a microfluidic device to investigate the resistance to GEM in PDAC cell cultures. The drug caused apoptosis in the PDAC cell lines, and its half-maximal effective concentration (EC_50_) was near nine-fold higher on the microchip culture when compared to a 2D culture (31 nM versus 277 nM, respectively).

#### 3.2.8. Liver

The liver is greatly affected by treatments since it is involved in drug metabolism. Some drugs are activated after the biotransformation in the liver [54]. Liang et al. [48] designed a microfluidic device (Figure 6c) with an integrated light addressable potentiometric sensor to monitor the pH and, consequently, the metabolism of the cell culture. To test the device, the authors used HepG2 cells and administered glucose and DOX on the device. As expected, the metabolism increased when higher doses of glucose (125 mM) were administered, while a significant decrease was registered when DOX in a 10 μM concentration was administered. Zuchowska et al. [55] developed a microfluidic device to test the effects of the drug 5-FU on HepG2 spheroids. Two concentrations of the drug, 0.5 mM and 1.0 mM were used on spheroids of different diameters. The authors reported a decrease in the drug resistance with the increase in spheroid diameter. Yu et al. [56] manufactured a microfluidic device to serve as a reliable, fast, and non-invasive tool for drug screening. The authors evaluated the cytotoxicity of DOX and CIS on HepG2 cells using the device through redox ratio imaging and using a traditional well-plate system. The reduction of cell viability with increasing drug concentration was registered on both methods. The IC_50_ of CIS was 174.6 μM and 280.8 μM on the redox ratio and MTS assay respectively, while the IC_50_ of DOX was 6.29 μM and 43.97 μM. 

Sun et al. [49] evaluated the anticancer effects of PTX and TPZ on a co-culture of hepatic healthy and cancer cells. The cells were placed on a microfluidic device designed to replicate the hypoxic microenvironment of a tumor as schematized in Figure 6d. The viability of the cells was significantly reduced when using TPZ, but not with PTX. The authors proposed that the activation of the healthy cells due to the co-culture and hypoxia conditions could have contributed to increasing the resistance to PTX.

#### 3.2.9. Kidney

**Figure 7:**
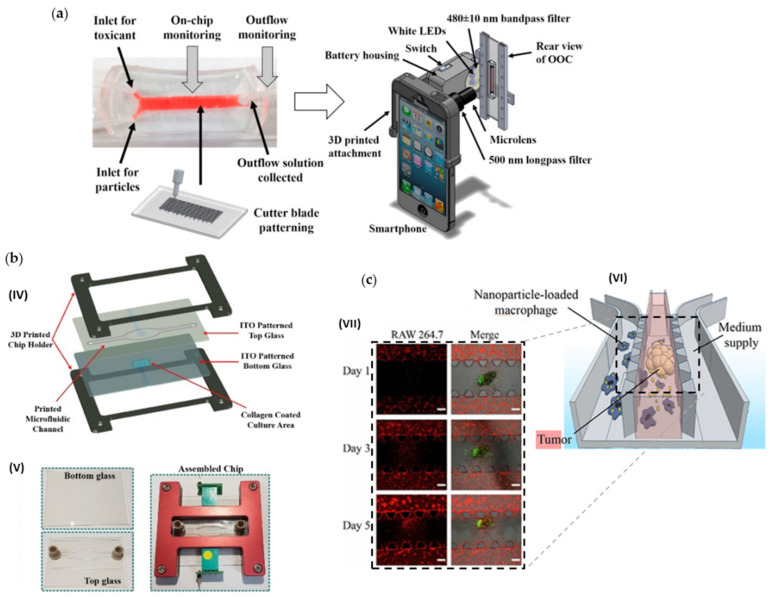
(**a**) Non-destructive, in situ monitoring of drug-induced nephrotoxicity on kidney-on-a-chip developed by Cho et al. [57] that quantifies immunocapture and immunoagglutination using a Smartphone-based fluorescence microscope. Adapted with permission from [57]. (**b**) Lung cancer-on-chip platform fabrication developed by Khalid et al. [59]: (IV) Expanded schematic view of the assembly of the 3D printed parts and (V) microfluidic chip images of the separate parts and final assembled chip. Adapted with permission from [59]. (**c**) Tumor-microenvironment-on-a-chip developed by Wang et al. [60] for drug-carrying macrophages and their delivery to tumors in vivo and in vitro: (VI) Schematic illustration, and (VII) Confocal images showing migration of RAW 264.7 cells (red) toward SKOV3 spheroids (green) in microfluidic channels on days 1, 3, and 5. Scale bars represent 200 μm. Adapted with permission from [60].

Cho et al. [57] manufactured an organ-on-a-chip (OoC) with a smartphone-based microscope attached to the in situ monitoring of the renal adenocarcinoma cell culture inside the device, Figure 7a. The evaluation of the nephrotoxicity or non-nephrotoxicity of a drug was performed by the detection of γ-glutamyl transpeptidase (GGT) on the outflow or on the apical border of the cells, respectively. The drugs CIS and glycine were tested in various concentrations. For glycine, the values of GGT on the outflow were low and were high on the cells, being roughly constant in both cases. For CIS, a similar behavior was noted for low concentrations, whereas in higher concentrations, the value of GGT on the cells decreased and, on the outflow, increased significantly. Virumbrales-Muñoz et al. [58] used a microfluidic device to recreate the blood vessel formation that occurs with the tumor growth. The authors used three patient-derived cancer cells on the model. The efficacy of anti-angiogenic drugs nintedanib and sirolimus were also evaluated. Though these drugs are not first-line treatments, they proved to be effective in reducing the number of angiogenic sprouts in one of the patients.

#### 3.2.10. Lung

Mani et al. [61] recreated the dynamic in vivo microenvironment of a non-small cell lung (NSCL) carcinoma on a microfluidic platform. The cytotoxic effects of the drugs erlotinib and NSC-750212 were investigated using 3D cultures on the chip and also 2D cultures in static and dynamic conditions. After the treatment with both drugs, the cell viability decreased significantly in both culture conditions. The drug NSC-750212 affected a higher percentage of cells when using 3D static conditions. Yang et al. [62] developed a lung-on-a-chip platform to test the effects of the anticancer drug gefitinib on a co-culture of NSCL cancer cells, human fetal lung fibroblasts, and human umbilical vein endothelial cells. The authors reported that the cancer cells had an influence on the endothelial cells by inducing apoptosis. After the treatment with gefitinib, the viability of the cancer cells was significantly reduced.

Khalid et al. [59] developed a lung cancer-on-chip device for cytotoxicity testing of anticancer drugs through the transepithelial electrical impedance (Figure 7b). The drugs DOX and docetaxel were tested on the platform. DOX led to higher cell death ratings with increasing concentration than docetaxel. The calculated IC_50_ was 6.791 μM for DOX and 0.137 nM for docetaxel. Dhiman et al. [63] combined hyperthermia with the co-culture of human amniotic membrane-derived mesenchymal stem cells (AMMSCs) with human lung carcinoma cells and applied anticancer peptide (P1) to the culture medium. The experiments were performed using a microfluidic device for the cell culture. AMMSCs contributed to the development of the tumor spheroids, but after heat treatment, reductions in cell proliferation and spheroid diameter were reported. Adding the anticancer peptide to the treatment increased the cytotoxic effects. Shin et al. [64] manufactured an open-cell culture platform with the assistance of bioprinting. Lung cancer cells were cultured on the device and perfused with the anticancer drug CIS. A dramatic reduction of the cell proliferation with the increase in the administered dose of CIS was reported. The registered IC_50_ of the drug for the cell type used was determined to be 98.91 μM.

Dhiman et al. [65] presented a microfluidic model with lung cancer cell spheroids trapped inside a compartment with collagen gel. The anticancer potential of tryptophan-rich peptide P1 was evaluated on the chip. High cytotoxicity was reported for the cancer cells, while low cytotoxicity was registered in human amniotic membrane mesenchymal stem cells cultures.

Kang et al. [66] tested the efficacy of the use of PTX, or coumarin-6 encapsulated gelatin–oleic conjugate nanoparticles on the treatment of lung cancer. The evaluation was performed using a microfluidic device to test different levels of shear stress. With the increase in shear stress, a reduction of the inhibitory concentration was reported. The values were also lower than the ones obtained in static conditions.

Meghani et al. [67] described an alveolus-epithelium-on-a-chip model for the evaluation and monitorization of the effects of pH-responsive zinc oxide quantum dots encapsulated by human serum albumin nanoparticles in cancer treatment. The cell viability was verified after the treatment with nanoparticles at concentrations of 10 and 50 μg/mL. After 24 h of treatment, 30% and 80% of cell death was registered for the lower and higher concentrations, respectively. Cell shrinkage was also reported.

#### 3.2.11. Breast

Du et al. [68] simulated the in vivo microenvironment of a breast tumor in a microfluidic chip by including interconnected regions for the cancer cells, normal cells, and vascular endothelial cells. The device was used to determine the invasiveness of the tumor and to test the efficacy of the drug PTX. The drug significantly reduced the cell viability and the average migration distance at the second and third days of treatment. Nguyen et al. [69] recapitulated the microenvironment of HER2+ breast cancer in a tumor-on-chip with a co-culture of four different cell populations: cancer, fibroblasts, endothelial and immune. The effects of the drug trastuzumab were evaluated with the chip. The drug led to an increase in the apoptosis rate and reduced the mitosis rate of the cancer cell line. Gokce et al. [70] compared the anticancer potential of the drug (R)-4′-methylklavuzon (KLA) with DOX on a lab-on-a-chip incorporated with a Matrigel for the culture of breast cancer cells. While testing using a 3D monoculture of cancer cells, both drugs showed similar effects. On the other hand, in a 3D tri-culture, DOX was more effective than KLA, but less selective, leading to the death of more normal mammary epithelial cells. Mun et al. [71] designed a microfluidic device to test photothermal therapies on co-cultures of cells. The authors conjugated folic acid and reduced graphene oxide (GO) to form a nanomaterial to target breast cancer cells, enhancing the therapeutic effect. After the treatment with the nanomaterial and near-infrared laser irradiation, the viability of the cancer cells on the microfluidic platform was reduced while the viability of the endothelial cells was maintained.

Guerrero et al. [72] developed a microfluidic device to evaluate the time- and dose-dependent effects of anticancer drugs on a 2D or 3D culture of cancer cells. The authors used breast cancer cells and tested the drugs DOX and GEM. The drugs were administrated separately and in sequence. The cells were more susceptible to the treatment with GEM than with DOX, but no significant difference was registered between the order of administration when using both. The cytotoxic effects were greater on the 2D culture than on the spheroid culture. Dong-Guo et al. [73] developed a microfluidic device where a nanoporous membrane was sandwiched between the culture chamber for the breast cancer cells and the reservoir chamber to mimic the in vivo vascularization environment surrounding the tumor. The authors compared the effect of DOX and PTX on the chip on a static 3D and 2D culture of cancer cells. Both drugs were increasingly cytotoxic with the increase in concentration, and the effects were greater on the 2D culture and smaller on the microfluidic device. Lanz et al. [74] used the microfluidic chip OrganoPlate^®^ to test three different anticancer drugs, PTX, olaparib, and CIS, on three types of breast cancer cells. The authors also compared the 3D model with a 2D cancer cell culture model. PTX and olaparib were administered together and separately. Olaparib had almost no effect on 2D and 3D cultures, while PTX led to a reduction of cell viability on both models. For the 2D, the maximum effect was obtained using the lowest concentration, while higher doses were required on the 3D model. The combination of both drugs led to a smaller effect on the 3D model when compared to the effect of PTX. CIS showed effects on one type of cancer cells at low concentrations on the 3D model and at higher concentrations on the 2D model.

Pradhan et al. [75] replicated the microvascular network present for in vivo breast cancers using two microfluidic devices. One with larger chambers for tumor cell culture and wider network designated with high perfusion chip (HPC), and the other with smaller chambers and network designated with low perfusion chip (LPC). The authors tested the cytotoxicity of two anticancer drugs, DOX and PTX, and verified a greater effect on the HPC than on the LPC. DOX was more effective than PTX, given that only DOX had some cytotoxic effect on the breast cancer cells of the LPC. The researchers also reported that both drugs presented a greater anti-tumor effect in static conditions than under flow conditions. Choi et al. [76] designed a breast cancer model that incorporates a set of compartments for the co-culture of mammary ductal epithelial cells, mammary fibroblasts, and breast tumor spheroids to mimic the microstructure of a breast ductal carcinoma. The device was used to evaluate the toxicity and efficacy of the anticancer drug PTX. The authors reported that PTX was cytotoxic for the cancer cells and prevented the enlargement of the tumor spheroids while not being cytotoxic for the healthy cells. Virumbrales-Muñoz et al. [77] developed a microfluidic device for the co-culture of breast cancer cells in a 3D model and endothelium in a 2D model. The registered proliferation of the cells and the oxygen gradients were similar to the ones observed in vivo. The authors tested the effects of TNF-related apoptosis-inducing ligand (TRAIL) on its soluble form and anchored to a large unilamellar vesicle (LUV). The conjugate LUV-TRAIL led to greater cell death of tumor cells while the endothelium cells were not affected.

Shirure et al. [78] presented a microfluidic device that replicates the tumor progression, including angiogenesis, cell migration, and intravasation. The authors tested the efficacy of the treatment with the chemotherapy drug PTX, using a cancer cell line and patient-derived tumor organoids (PTDO). Vascular density and size of the tumor from the cancer cell line were reduced after the treatment. The vascular density was not affected on the PTDO, but the tumor growth was reduced after the treatment. Humayun et al. [79] established a microfluidic model to study the metastatic potential of breast cancer cell lines. The device presented a central microchannel that replicates a tubular endothelial vessel where the cancer cells were cultured. On both sides of the central channel were two other channels filled with media. The authors reported a correlation between an increase in the secretion of IL-6, IL-8, and MMP-3 with the invasiveness of the tumor. The therapeutic agents, tocilizumab, reparixin, and UK-356618, were tested since they inhibit the secretion of those factors. The cytotoxicity for epithelial cells was firstly evaluated and for values above 500 μg/mL, 50 μM, and 6 μM for reparixin, tocilizumab, and UK-356618 respectively, the viability was highly diminished. Consequently, lower concentrations were used. The secretion was significantly reduced after the treatment, being the effect more pronounced with the use of combined treatment than with individual drugs. The combination of the three drugs also significantly reduced the extravasation behavior of the cancer cells.

#### 3.2.12. Reproductive System

Flont et al. [80] developed a microfluidic device to culture multilayers of patient-derived ovarian cancer cells. The efficacy of anticancer photodynamic therapy (PDT) was evaluated using the chip with free and nanoencapsulated meso-tetrafenylporphyrin as the photosensitizers. The photosensitizers contributed to increasing the efficacy of the PDT, especially under the nanoencapsulated form. Nevertheless, the photosensitizers must be used in small concentrations since they are also toxic for healthy cells.

Wang et al. [60] developed a tumor-microenvironment-on-a-chip device for the co-culture of macrophages and ovarian adenocarcinoma spheroids in a 3D gel matrix. The macrophages served as carriers of PTX-loaded polymer nanoparticles. The loading of the macrophages with the nanoparticles did not cause the reduction of the cell viability, and the cytotoxicity on the cancer cells was higher than when administering only the nanoparticles, Figure 7c.

#### 3.2.13. Multi-Organ Systems

**Figure 8:**
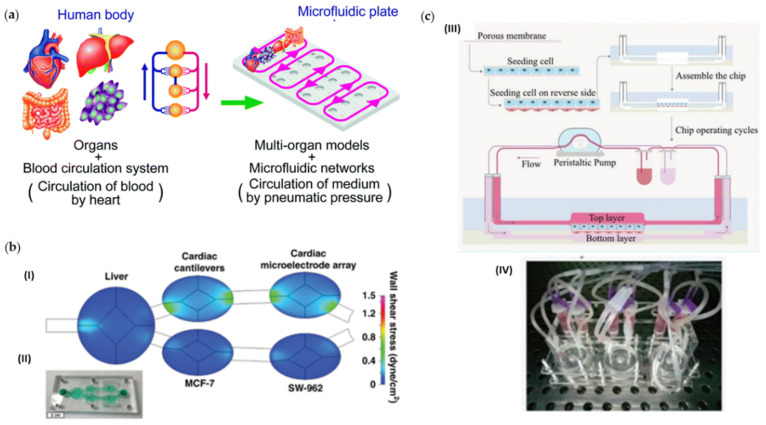
Multi-organ systems. (**a**) Concept of a multi-throughput multi-organ-on-a-plate system and the culture devices in the assembled state and with the lid removed fabricated by Satoh et al. [82]. Adapted from [83]. (**b**) Five-chamber multi-organ system platform used by McAleer et al. [84] to evaluate the efficacy and off-target toxicity of anticancer drugs: (I) Computational fluid dynamics modeling and (II) the fabricated microfluidic system. Adapted with permission from [83]. (**c**) Illustration of the chip assembly and operation developed by Liu et al. [85]: (III) Cells were sequentially seeded on the front and back of the porous membrane prior to chip assembling; (IV) Picture of the entire system of the chip devices operated by a peristaltic pump. Adapted with permission from [85].

Xu et al. [81] designed a BBB model to study the effects of the barrier on the migration of metastasis from cancerous cells, namely lung, breast, and melanoma cells. The authors reported that the interaction of those cells with BBB astrocytes contributed to the permeability of the barrier to the cells. Some chemotherapeutic agents were also tested regarding their cytotoxicity against the tumorous cells and their ability to traverse the BBB. Of the tested drugs, only TMZ showed anti-tumor effects with and without the BBB, leading to the apoptosis of 77% of glioma cells. CBP, CIS, 5-Fu, nedaplatin (NDP), and GEM only were effective in the absence of the barrier, while FTO and ifosfamide (IFO) showed no effect at all since those compounds need to be metabolized by the liver. Satoh et al. [82] reported a multi-OoC system to simultaneously test the effect of anticancer drugs on cancer and healthy cells. The platform was composed of a two-organ system, with a culture of colorectal cancer cells and healthy liver cells, and a four-organ system, with the same cells and also intestine and connective tissue models, Figure 8a. On the first system, the prodrug capecitabine (CAP) was tested, while on the second was the prodrug tegafur. The metabolite of both drugs is 5-FU. The growth of colorectal cancer was inhibited in the cultures connected to the liver model. Some inhibition was also registered on the connective tissue model, while no significant effects were reported on the remaining models.

Kimura et al. [86] described a microfluidic model to mimic the small intestine–liver interactions in vivo. The authors cultured lung cancer cells together with small intestine and liver cancer cells on the platform and evaluated the anticancer activity of three drugs, epirubicine, irinotecan (CPT-11), and cyclophosphamide (CP). The treatment led to the reduction of the lung cancer cells viability. The effect was more pronounced when the cells were cultured with liver cells than with the intestine cell line.

McAleer et al. [84] developed a pumpless multi-organ–on–a–chip system for the prediction of on-target efficacy and off-target toxicity of therapeutic drugs, Figure 8b. The authors evaluated two configurations of the system. On the first configuration, primary human hepatocytes and two cancer-derived human bone marrow cell lines were cultured on the device. The antileukemia drugs diclofenac and imatinib inhibited tumor cell growth. However, diclofenac also reduced the viability of the hepatic cells. On the second configuration, a non–multidrug-resistant breast cancer line, a multidrug-resistant vulva cancer line, induced pluripotent stem cell-derived cardiomyocytes, and primary hepatocytes were cultured on the device. The anticancer drug tamoxifen was tested alone and together with verapamil. In both cases, effects on the conduction velocity, beat frequency, and contractile force of the cardiac cells were registered though no effects on cell viability were reported. Tamoxifen affected the viability of breast cancer cells only after metabolization by the liver cells and affected the vulva cancer cells only when combined with verapamil. Jie et al. [87] developed a biomimetic intestine–liver glioblastoma microfluidic platform to assess the therapeutic potential of several drug combinations for glioblastoma. The intestine and liver constructs of the device served to mimic the delivery and metabolization of the drugs, respectively. The tested drugs were CP, CPT-11, and TMZ. The metabolites of the drugs induced apoptosis of the glioblastoma cells. A CPT-11 and TMZ combination showed better results than the individual drugs and CPT-11 and CP, and TMZ and CP combinations. Ma et al. [88] evaluated the cytotoxicity of anticancer drugs, as well as their metabolism on a microfluidic device with two connected chambers. In one chamber, glioblastoma multiforme brain cancer cells were cultured, while on the other, liver cells with different cytochrome P450 subtypes were cultured. The drugs TMZ and IFO were perfused through the device. The effect of IFO on the glioblastoma cells was dependent on the type of hepatic cells present. The effect of TMZ was lower than IFO and more pronounced on 2D cultures than on the 3D culture of the device.

Li et al. [89] presented a microfluidic platform to evaluate the nephrotoxicity of cancer drugs. The device was composed of two compartments for the cell culture, separated by a porous membrane. Hepatic cancer cells and renal cells were cultured in the chambers of the device and perfused with the drugs IFO and verapamil. Nephrotoxic effects due to the metabolites of the drugs were registered. In another study [90], the researchers presented a device for the culture of four different cell types, liver and gastric cells, breast, and lung cancer cells. The effects of the prodrug CAP on the different cells were evaluated. The metabolite of the drug, 5-FU, generated by activation on the liver, contributed to the cytotoxic effects of the drug on the cancer cells. On the other hand, no effects were reported on the gastric cells. Chen et al. [54] presented a microfluidic system that incorporated a liver-on-a-chip (LOC) connected to a tumor-on-chip to evaluate the effects of statin and its respective metabolites on healthy and tumor cell lines. The expression of metabolic markers on the LOC increased when connected to the cancer culture. Metabolites of simvastatin showed cytotoxic effects on human liver cancer and cancer prostatic cells, while no significant effects were reported on primary fibroblasts and hepatocytes. Rajan et al. [91] reported a multi-organoid system to assess the toxicity and efficiency of drugs on several 3D tissue organoids. The device was constituted by interconnected chambers with a hyaluronic acid hydrogel for the seeding of spheroids from the corresponding cell line. On the first model, the authors used a system with three chambers for liver, cardiac, and lung models. The drug CAP was administered on the liver chamber, and cytotoxic effects due to the metabolite 5-FU were reported on the cardiac model. On a second model, the authors also added chambers for endothelium, brain, and testes models. The administration of the anticancer drug IFO on the system through the liver construct led to neurotoxicity due to the formed metabolites.

Skardal et al. [92] designed a microfluidic platform with several interconnected modules for the culture and growth of vascular, cardiac, colon, lung, brain, testis, and liver cells. The effects of several toxic and non-toxic drugs on the different models were evaluated. Two of the tested drugs were the chemotherapeutical agents, CAP and CP. The presence of the liver module affected the toxicity of CAP since it had to be metabolized into its active form. Consequently, in the absence of the liver module, no toxicity on healthy and cancer cells was registered. With the incorporation of the liver module, toxicity on the cardiac and lung modules was reported. Despite not needing to be metabolized to become active, CP metabolites also caused toxicity on the cardiac and lung modules. Weng et al. [93] developed a microfluidic chip for the simultaneous culture of cancer cell spheroids and human induced pluripotent stem cardiomyocytes and endothelial cells. The calcium transients on the cardiac tissue were monitored in real-time, so the cardiotoxic effects of anticancer drugs were evaluated on the platform. A culture of colon cancer cells was seeded on the device and treated with DOX and oxaliplatin. At concentrations near the IC_50_, DOX diminished the spontaneous beating rate, while that effect was only reported for concentrations of oxaliplatin above the IC_50_. Kamei et al. [94] also integrated human healthy heart cells and liver cancer cells on a microfluidic chip to test the toxic effects of DOX. The drug reduced the viability of the cancer cells and did not affect the cardiac cells. However, the metabolites derived from the drug showed significant toxic effects on the cardiac cells. 

Jie et al. [95] designed a microfluidic platform for the simulation of the absorption, metabolism, and activity of anticancer drugs. On the chip, a channel for the culture of hepatic cancer cells was separated by a polycarbonate semipermeable membrane from another channel with a culture of colorectal cancer cells. The first channel was also connected to another channel with a culture of glioblastoma cells. CPT-11 was administered through the colorectal channel, and the cytotoxic effects were studied. The viability of the three cell lines was reduced with the increasing concentration of the drug. The reduction was less significant in dynamic conditions than in static conditions. Jie et al. [96] developed a microfluidic device with two layers for different cell cultures to study the metabolism and adsorption of anticancer drugs. Adenocarcinoma and liver cancer cells were seeded on the device and treated with genistein and dacarbazine. For concentrations below 100 μg/mL, no significant inhibition effect was registered, while for concentrations above 250 μg/mL, the liver cancer cells underwent apoptosis. Liu et al. [97] introduced a microfluidic platform with pneumatic microstructures for the dynamic throughput and control of 3D tumor cultures. The authors tested the apoptotic effects of vinorelbine and GEM on mono- and co-cultures of three different cancer cell lines. The used cell lines were liver carcinoma, glioma, and gastric carcinoma. The drugs led to the increase in a lysosomal enzyme involved in the apoptotic pathway with increasing concentration. Monocultures showed greater resistance to the drugs than co-cultures.

LaValley et al. [98] designed a pumpless body-on-a-chip microfluidic device for the test of anticancer drugs on several cell lines. The device presented different compartments with collagen hydrogels for the culture of colon cancer spheroids, hepatocytes, and promyeloblasts. The combination of the anticancer drug tegafur with uracil was administered to the system. The treatment induced the death of the colon cancer cells and, consequently, reduced the size of the spheroids. Toxic effects were also reported on the promyeloblasts cell line. Hübner et al. [99] adapted a commercially available multi-organ chip device for the culture of lung cancer microtissues and human skin in two connected culture compartments. The efficacy of the drug anti-EGFR-antibody cetuximab was tested on the platform. The drug led to an increase in the expression of a pro-apoptotic related gene in the cancer microtissues and also to the elimination of proliferative keratinocytes on the skin. 

Liu et al. [85] studied the cytotoxic effects of that ginsenosides compound K (CK) on several cell lines, both in single and multi-organ chips, Figure 8c. Intestinal and liver carcinoma cell lines and vascular and kidney cell lines were seeded on the chips. No toxic effects were reported when using low concentrations of the drug. However, higher concentrations led to toxic effects in all cell lines. On the multi-organ chip, the administration of the drug was performed through the intestinal module, which led to the accumulation of 70% of the drug in that region, and only a small percentage was found on the remaining culture chambers. Zuchowska et al. [100] presented a microfluidic device for the simultaneous culture of cancer cells in a monolayer (2D model) and in spheroids (3D model). The authors used the device to test the cytotoxic effects of GO on colon, liver, and breast cancer cells. No major effect on the cell viability was registered, being only slightly reduced on the breast and liver cancer spheroids. The 2D models showed more resistance to GO than the 3D models.

### 3.3. Summary Results from 2D vs. 3D Culture Systems in Revision

A summary of the most important features of the previously discussed OoC and the comparison between types of cell cultures is presented in Table 1. Several authors have sought to replicate not only the tumor microenvironment but also specific characteristics of the system that was being reproduced, as described in [28,62,76]. A few studies also replicate patient-specific features and treatment responses [30]. The different OoC devices were used for model validation, and some contributed to the test of novel treatments such as magnetic microwires [34] and drug-loaded nanocarriers [28]. Differences regarding drug resistance between 3D and 2D cultures were noted in several studies, showing the importance of OoC devices to prevent unnecessary testing of inefficient drugs using animal models. The 3D dynamic cultures provided by the OoC improved some of the features that were lacking in the static culture conditions, such as continuous and well distribute medium supply, waste removal, and oxygen perfusion. Furthermore, the integration of multiple functions such as sampling, capture, lysis, imaging, and detection within one platform led to the greatest developments in the OoC applications, as summarized in Table 1.

## 4. Conclusions

OoC devices have been proving their potential as low-cost, easy to replicate, and reliable tools for a more detailed study of disease progression mechanisms and treatment effects. Several studies noted the difference between the effects of therapeutic drugs on 2D and 3D cultures. The dose to the same effects tends to be higher and closer to the observed in animal models for the later cultures.

On the other hand, 3D cultures do not fully represent the in vivo physiological conditions and structures of both healthy and pathologic tissues. Several authors recreated the vascular network that is observed in tumor microenvironments on the OoC. Those models allowed the study of the migration and evasion of other tissues by tumor metastasis, the evaluation of the effects of anti-angiogenic drugs, and the influence of the vascular network on the drug efficacy. Some authors reported an increase in the drug efficacy on models with the vascular network since more regions of the tumor were subjected to the drug. Other authors reported less efficiency of the tested drugs due to the increased supply of oxygen and nutrients provided by the vessels.

In several studies, the combination of cultures of different organ cells was explored to better reproduce the in vivo environment. Some anticancer drugs require metabolization by the liver to become active, so to evaluate the effect for the corresponding dosage, it is necessary to consider that process in the in vitro studies. Conversely, some of the metabolites generated by liver metabolization can be toxic for non-target tissues, for example, the cardiac muscle. The intestinal track also has a great influence on the dose-dependent effects of drugs. Most treatments are administered through that system, which might lead to the accumulation of drugs on the intestinal tissues.

Overall, recent developments on microfluidic devices have been successful in replicating in vitro some of the most relevant features found within in vivo systems. The possibility of using patient cells contributes to a more precise evaluation of which treatment options are more effective and what are the mechanisms related to the resistance to certain options. However, most of the current studies still use commercial cell lines and standard therapies as a proof-of-concept for their device. Few studies reported the influence of the materials used on the adsorption of the drugs by the OoC materials. Further improvements are still required until a human-on-a-chip becomes a standard disease evaluation tool. Nevertheless, several studies have already contributed towards reaching that goal.

## Figures and Tables

**Table 1:** Features and comparison results between cell culture systems presented on representative references by organ models.

*Organ/System*	Features	Research Goal	Evaluated Drugs	Comparison Results between Cell Culture Systems	Representative Ref.
*Brain and Nervous system*	Mimic of BBB and glioblastoma microenvironment	NPs nanodelivery of cancer drug for glioblastoma treatment; validation of the OoC as a patient-specific cancer model	Antibody-functionalized nutlin-loaded nanostructured lipid carriers; chemoradiation using TMZ, CIS, O^6^BG and MX	Greater drug resistance in 2D cultures than in 3D cultures	[28,30]
*Bone*	Microstructures for increased liquid mixing and cell-treatment interaction	Novel osteosarcoma treatment assessment;clinical drug validation	Fe77B10Si10C3 glass-coated amorphous magnetic microwires, MTX based treatments	Sedimentation of nanoparticles in traditional assays with static conditions lead to problems such as cell death being caused by undesired mechanisms	[33,34]
*Lymphatic System*	Incorporation of different types of cells to mimic tumor microenvironment	Novel approach for creation of 3D tumor-stromal-immune cell spheroids	Lenalidomide	Cell death and reduction of proliferation higher in 2D cultures than in the 3D culture	[35]
*Angiogenesis*	Hydrogel that leads to angiogenic sprouting patterns, pores to simulate capillaries, continuous fluid perfusion	Model validation for replication of tumor vasculature	Apatinib, vandetanib, linifanib, cabozantinib, cetuximab, bevacizumab	The effectiveness of some tested drugs was superior in 2D monolayer cultures while the opposite was noticed on others when compared to 3D cultures.Vasculature is not mimicked in 2D cultures	[37,43]
*Pancreas*	Culture of cells with different phenotypes to mimic tumor microenvironment and intra-tumoral heterogeneity; endothelium-mimicking membrane	Pancreatic ductal adenocarcinoma model validation for drug evaluation	GEM	IC50 and EC50 values of tested drugs were higher for the 3D culture than for the 2D culture	[52,53]
*Lung*	Layers separated by porous membrane to simulate blood–air interface	Lung cancer model validation for real-time drug effect evaluation	DOX and docetaxel, gefitinib	More cells were affected by therapeutic drug in 3D static culture than in 3D dynamic culture or 2D static and dynamic cultures	[59,62]
*Breast*	Microchannels separated by a thin ECM-derived membrane to replicate the human mammary duct	Evaluation of specific cellular signaling; breast ductal carcinoma model validation for drug evaluation	Tocilizumab, reparixin, UK-356618, PTX	Cytotoxic effects of therapeutic drugs greater on the 2D culture than on the 3D culture	[76,79]
